# 6-Amino­nicotinamide

**DOI:** 10.1107/S1600536812031224

**Published:** 2012-07-14

**Authors:** Lerato P. Ntsala, Andreas Lemmerer

**Affiliations:** a Molecular Sciences Institute, School of Chemistry, University of the Witwatersrand, Private Bag 3, PO WITS 2050, Johannesburg, South Africa

## Abstract

In the title compound, C_6_H_7_N_3_O, the amide group is rotated such that the carbonyl O atom is *syn* to the pyridine N atom, with an O—C—C—C torsion angle of −23.55 (18)°. The crystal packing involves four hydrogen bonds of the types N—H⋯N and N—H⋯O. Two separate centrosymmetric rings are formed using N—H⋯N and N—H⋯O hydrogen bonds that result in a ribbon of 6-aminonicotinamide molecules, joined by the amide and amine functional groups. The remaining two hydrogen bonds are used to generate a three-dimensional packing arrangement.

## Related literature

For pharmacological activity, see: Street *et al.* (1997 ▶); Budihardjo *et al.* (2000 ▶). For structurally related compounds, see: Miwa *et al.* (1999 ▶); Li *et al.* (2011 ▶).
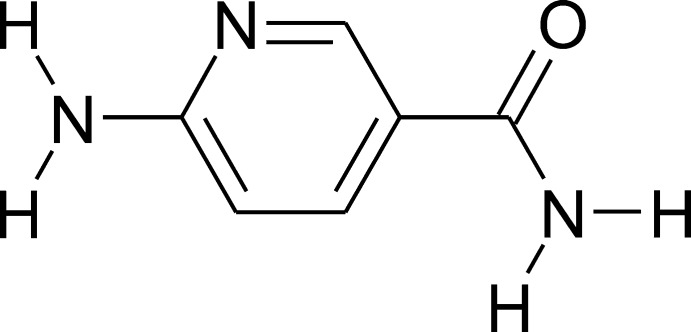


## Experimental

### 

#### Crystal data

C_6_H_7_N_3_O
*M* *_r_* = 137.15
Monoclinic, 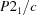

*a* = 14.3483 (6) Å
*b* = 4.8143 (2) Å
*c* = 9.6685 (4) Å
β = 99.215 (2)°
*V* = 659.25 (5) Å^3^
*Z* = 4
Mo *K*α radiation
μ = 0.1 mm^−1^
*T* = 173 K
0.27 × 0.25 × 0.2 mm

#### Data collection

Bruker APEXII CCD area-detector diffractometer
Absorption correction: multi-scan (*SADABS*; Sheldrick, 1996 ▶) *T*_min_ = 0.974, *T*_max_ = 0.980
4078 measured reflections
1582 independent reflections
1300 reflections with *I* > 2σ(*I*)
*R*_int_ = 0.037

#### Refinement

*R*[*F* ^2^ > 2σ(*F* ^2^)] = 0.040
*wR*(*F* ^2^) = 0.113
*S* = 1.03
1582 reflections
107 parameters
H atoms treated by a mixture of independent and constrained refinement
Δρ_max_ = 0.33 e Å^−3^
Δρ_min_ = −0.21 e Å^−3^

### 

Data collection: *APEX2* (Bruker, 2005 ▶); cell refinement: *SAINT-Plus* (Bruker, 2004 ▶); data reduction: *SAINT-Plus* and *XPREP* (Bruker 2004 ▶); program(s) used to solve structure: *SHELXS97* (Sheldrick, 2008 ▶); program(s) used to refine structure: *SHELXL97* (Sheldrick, 2008 ▶); molecular graphics: *ORTEP-3 for Windows* (Farrugia, 1997 ▶) and *Mercury* (Macrae *et al.*, 2006 ▶); software used to prepare material for publication: *WinGX* (Farrugia, 1999 ▶) and *PLATON* (Spek, 2009 ▶).

## Supplementary Material

Crystal structure: contains datablock(s) global, I. DOI: 10.1107/S1600536812031224/fj2584sup1.cif

Structure factors: contains datablock(s) I. DOI: 10.1107/S1600536812031224/fj2584Isup2.hkl

Supplementary material file. DOI: 10.1107/S1600536812031224/fj2584Isup3.mol

Supplementary material file. DOI: 10.1107/S1600536812031224/fj2584Isup4.cml

Additional supplementary materials:  crystallographic information; 3D view; checkCIF report

## Figures and Tables

**Table 1:** Hydrogen-bond geometry (Å, °)

*D*—H⋯*A*	*D*—H	H⋯*A*	*D*⋯*A*	*D*—H⋯*A*
N1—H1*A*⋯O1^i^	0.888 (18)	2.021 (19)	2.8933 (15)	167.2 (15)
N1—H1*S*⋯O1^ii^	0.907 (18)	1.997 (19)	2.9024 (14)	176.0 (16)
N3—H3*S*⋯N2^iii^	0.915 (18)	2.125 (19)	3.0322 (15)	170.9 (15)
N3—H3*A*⋯N3^iv^	0.875 (17)	2.363 (17)	3.2083 (16)	162.7 (15)

Symmetry codes: (i) 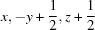; (ii) 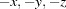; (iii) 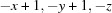; (iv) 
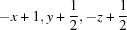
.

**Fig. 1.**
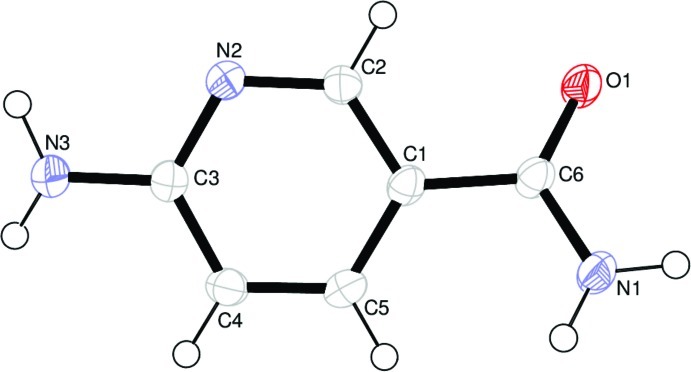
The asymmetric unit of (I) showing the atomic numbering scheme. Displacement ellipsoids are shown at the 50% probability level.

**Fig. 2.**
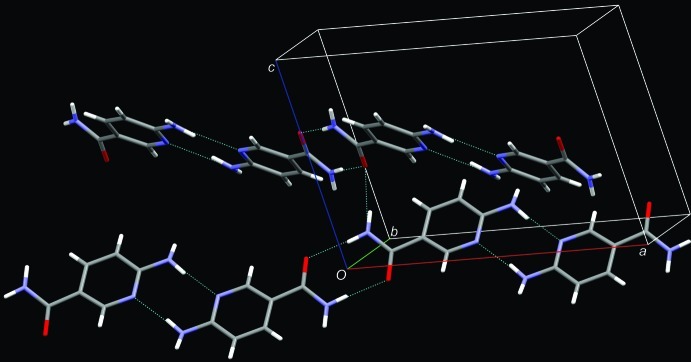
View of the hydrogen-bonded ribbons formed by the centrosymmetric dimers of the amine and amide functional groups of (I). H atoms not involved in hydrogen bonding are omitted for clarity. Intermolecular N—H···N and N—H···O hydrogen bonds are shown as dashed blue lines.

**Fig. 3.**
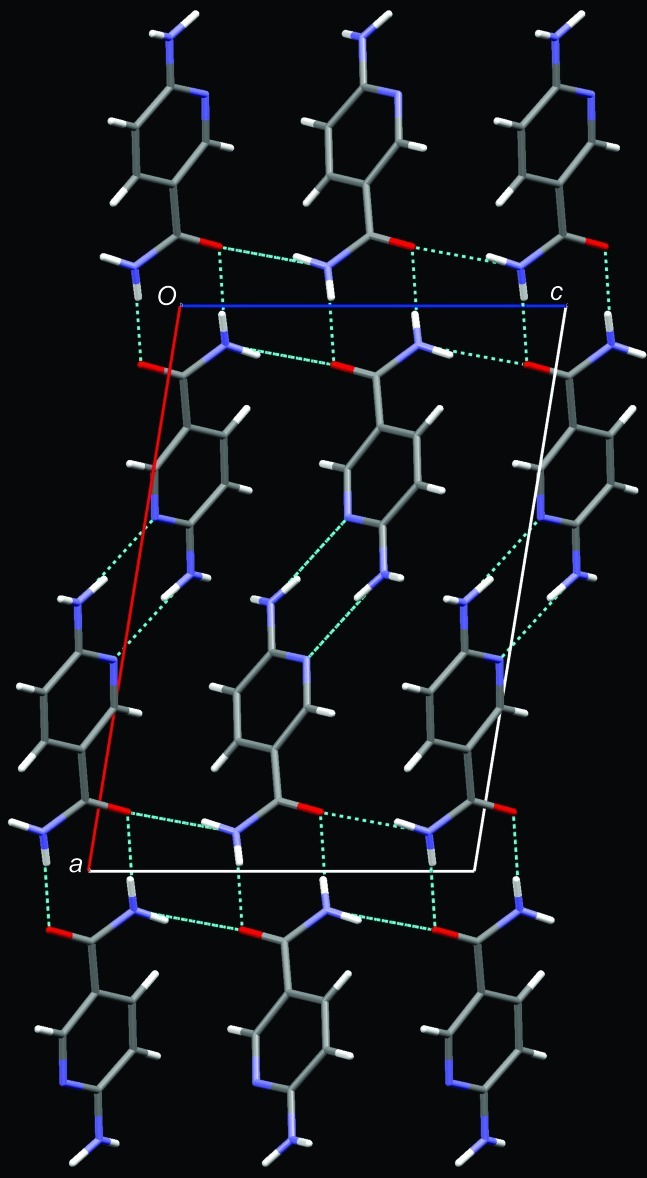
Packing diagram of (I). The 3-D network of hydrogen bonds is shown. H atoms not involved in hydrogen bonding are omitted for clarity.

## supplementary crystallographic information

### Comment

The title compound, 6-aminonicotinamide, and commonly abbreviated to 6AN, is a potent inhibitor of the pentose phosphate pathway (PPP) enzyme, 6PG dehydeogenase, which is an important step in the synthesis of NADPH and ribose units required for biosynthesis and DNA repair (Street *et al.*, 1997). Inhibition of this enzyme by 6-AN leads to accumulation of 6PG. In addition, it has been used in preclinical trials to enhance the effectiveness of cisplatin (Budihardjo *et al.*, 2000). To date, its crystal structure has not been reported.

The asymmetric unit of (I) consists of one molecule of 6AN on a general position and Fig. 1 shows the atomic numbering scheme. There are two single bonds allowing for torsional freedom, the amide group and the amine group, both relative to the pyridine ring. The torsion angle O1—C6—C1—C2 of -23.55 (18) is indicative of a *syn* conformation of the carbonyl to the pyridine N atom. This conformation is opposite to that of any of the polymorphs of the parent unsubstituted compound, nicotinamide, where the torsion angle ranges from -157.6 (1) to 167.1 (1)° (Miwa *et al.*, 1999; Li *et al.*, 2011). The hydrogen bonding of (I) makes use of all four hydrogen atom donors, two on the amide group and two on the amine. The *syn* H on the amide forms a homomeric centrosymmetric dimer using N1—H1S···O1 hydrogen bonds, while the H atom *syn* to the pyridine forms a second centrosymmetric dimer by hydrogen bonding to the pyridine, using N3—H3S···.N2 hydrogen bonds. The combination of these two dimers results in 1-D ribbons extended along the [110] direction. These ribbons are joined by N—-H···O hydrogen bonds from the anti H on the amide group (Fig. 2). Ultimately a 3-D arrangement results (Fig. 3), further supported by the N3—H3A···N3 hydrogen bond from the second H atom on the amine (not shown for clarity in Fig. 3).

### Experimental

Crystals of (I) where grown by dissolving 0.200 g (1.46 mmol) in 10 ml of AR-grade methanol and allowing for slow evaporation at room temperature over a few days. Cube-like colourless crystals where obtained.

### Refinement

The C-bound H atoms were geometrically placed (C—H bond lengths of 0.95 for aromatic CH) and refined as riding with *U*_iso_(H) = 1.2*U*_eq_(C). The N-bound H atoms were located in the difference map and coordinates refined freely together with their isotropic thermal parameters.

### Crystal data

**Table:**

C_6_H_7_N_3_O	*F*(000) = 288
*M**_r_* = 137.15	*D*_x_ = 1.382 Mg m^−^^3^
Monoclinic, *P*2_1_/*c*	Mo *K*α radiation, λ = 0.71073 Å
Hall symbol: -P 2ybc	Cell parameters from 1574 reflections
*a* = 14.3483 (6) Å	θ = 2.9–28.4°
*b* = 4.8143 (2) Å	µ = 0.1 mm^−^^1^
*c* = 9.6685 (4) Å	*T* = 173 K
β = 99.215 (2)°	Cube, colourless
*V* = 659.25 (5) Å^3^	0.27 × 0.25 × 0.2 mm
*Z* = 4	

### Data collection

**Table:**

Bruker APEXII CCD area-detector diffractometer	1300 reflections with *I* > 2σ(*I*)
ω scans	*R*_int_ = 0.037
Absorption correction: multi-scan (*SADABS*; Sheldrick, 1996)	θ_max_ = 28°, θ_min_ = 2.9°
*T*_min_ = 0.974, *T*_max_ = 0.980	*h* = −18→18
4078 measured reflections	*k* = −6→6
1582 independent reflections	*l* = −11→12

### Refinement

**Table:**

Refinement on *F*^2^	0 restraints
Least-squares matrix: full	H atoms treated by a mixture of independent and constrained refinement
*R*[*F*^2^ > 2σ(*F*^2^)] = 0.040	*w* = 1/[σ^2^(*F*_o_^2^) + (0.0577*P*)^2^ + 0.1618*P*] where *P* = (*F*_o_^2^ + 2*F*_c_^2^)/3
*wR*(*F*^2^) = 0.113	(Δ/σ)_max_ < 0.001
*S* = 1.03	Δρ_max_ = 0.33 e Å^−^^3^
1582 reflections	Δρ_min_ = −0.21 e Å^−^^3^
107 parameters	

### Special details

**Table:**

Experimental. Absorption corrections were made using the program *SADABS* (Sheldrick, 1996)
Geometry. All e.s.d.'s (except the e.s.d. in the dihedral angle between two l.s. planes) are estimated using the full covariance matrix. The cell e.s.d.'s are taken into account individually in the estimation of e.s.d.'s in distances, angles and torsion angles; correlations between e.s.d.'s in cell parameters are only used when they are defined by crystal symmetry. An approximate (isotropic) treatment of cell e.s.d.'s is used for estimating e.s.d.'s involving l.s. planes.

### Fractional atomic coordinates and isotropic or equivalent isotropic displacement parameters (Å^2^)

**Table:**

	*x*	*y*	*z*	*U*_iso_*/*U*_eq_	
C1	0.21465 (8)	0.3830 (3)	0.06966 (12)	0.0216 (3)	
C2	0.28897 (8)	0.3112 (3)	0.00180 (12)	0.0227 (3)	
H2	0.279	0.1654	−0.0651	0.027*	
C3	0.38732 (8)	0.6424 (2)	0.11660 (12)	0.0212 (3)	
C4	0.31587 (9)	0.7304 (3)	0.19125 (13)	0.0246 (3)	
H4	0.3271	0.878	0.257	0.03*	
C5	0.22979 (9)	0.5994 (3)	0.16746 (13)	0.0242 (3)	
H5	0.1808	0.6552	0.217	0.029*	
C6	0.12477 (8)	0.2249 (3)	0.03728 (12)	0.0235 (3)	
N1	0.06896 (8)	0.2202 (3)	0.13454 (12)	0.0315 (3)	
H1S	0.0150 (13)	0.121 (3)	0.1119 (18)	0.037 (4)*	
H1A	0.0869 (12)	0.292 (3)	0.219 (2)	0.036 (4)*	
N2	0.37410 (7)	0.4326 (2)	0.02370 (11)	0.0232 (3)	
N3	0.47474 (8)	0.7598 (2)	0.14074 (13)	0.0275 (3)	
H3S	0.5162 (12)	0.710 (4)	0.0824 (19)	0.037 (4)*	
H3A	0.4816 (12)	0.918 (3)	0.1855 (18)	0.034 (4)*	
O1	0.10436 (6)	0.0996 (2)	−0.07631 (9)	0.0295 (3)	

### Atomic displacement parameters (Å^2^)

**Table:**

	*U*^11^	*U*^22^	*U*^33^	*U*^12^	*U*^13^	*U*^23^
C1	0.0207 (5)	0.0269 (6)	0.0175 (5)	−0.0015 (4)	0.0042 (4)	0.0035 (4)
C2	0.0232 (6)	0.0257 (6)	0.0194 (6)	−0.0029 (5)	0.0045 (4)	−0.0017 (5)
C3	0.0206 (6)	0.0219 (6)	0.0208 (6)	−0.0007 (4)	0.0027 (4)	0.0028 (4)
C4	0.0275 (6)	0.0239 (6)	0.0229 (6)	−0.0008 (5)	0.0058 (5)	−0.0034 (5)
C5	0.0234 (6)	0.0281 (6)	0.0223 (6)	0.0026 (5)	0.0078 (5)	0.0010 (5)
C6	0.0203 (6)	0.0320 (6)	0.0185 (6)	−0.0012 (5)	0.0039 (4)	0.0023 (5)
N1	0.0246 (6)	0.0497 (7)	0.0219 (6)	−0.0122 (5)	0.0086 (4)	−0.0067 (5)
N2	0.0219 (5)	0.0260 (5)	0.0224 (5)	−0.0017 (4)	0.0061 (4)	−0.0017 (4)
N3	0.0229 (6)	0.0275 (6)	0.0329 (6)	−0.0045 (4)	0.0068 (4)	−0.0066 (5)
O1	0.0250 (5)	0.0451 (6)	0.0190 (5)	−0.0094 (4)	0.0057 (3)	−0.0040 (4)

### Geometric parameters (Å, º)

**Table:**

C1—C2	1.3824 (17)	C4—H4	0.95
C1—C5	1.4004 (17)	C5—H5	0.95
C1—C6	1.4870 (16)	C6—O1	1.2461 (15)
C2—N2	1.3401 (15)	C6—N1	1.3293 (16)
C2—H2	0.95	N1—H1S	0.907 (18)
C3—N2	1.3449 (15)	N1—H1A	0.888 (18)
C3—N3	1.3614 (15)	N3—H3S	0.915 (18)
C3—C4	1.4100 (17)	N3—H3A	0.875 (17)
C4—C5	1.3730 (17)		
			
C2—C1—C5	117.29 (11)	C4—C5—H5	120.2
C2—C1—C6	118.75 (11)	C1—C5—H5	120.2
C5—C1—C6	123.95 (11)	O1—C6—N1	122.15 (12)
N2—C2—C1	124.66 (11)	O1—C6—C1	120.45 (11)
N2—C2—H2	117.7	N1—C6—C1	117.39 (11)
C1—C2—H2	117.7	C6—N1—H1S	115.2 (11)
N2—C3—N3	116.99 (11)	C6—N1—H1A	121.9 (11)
N2—C3—C4	122.08 (11)	H1S—N1—H1A	122.5 (16)
N3—C3—C4	120.87 (11)	C2—N2—C3	117.43 (10)
C5—C4—C3	118.99 (11)	C3—N3—H3S	117.2 (11)
C5—C4—H4	120.5	C3—N3—H3A	118.4 (11)
C3—C4—H4	120.5	H3S—N3—H3A	120.1 (15)
C4—C5—C1	119.53 (11)		
			
C5—C1—C2—N2	0.43 (19)	C2—C1—C6—O1	−23.55 (18)
C6—C1—C2—N2	−178.45 (11)	C5—C1—C6—O1	157.66 (12)
N2—C3—C4—C5	−0.61 (19)	C2—C1—C6—N1	155.44 (12)
N3—C3—C4—C5	−177.58 (11)	C5—C1—C6—N1	−23.36 (18)
C3—C4—C5—C1	−0.24 (18)	C1—C2—N2—C3	−1.24 (18)
C2—C1—C5—C4	0.34 (18)	N3—C3—N2—C2	178.40 (11)
C6—C1—C5—C4	179.15 (11)	C4—C3—N2—C2	1.31 (18)

### Hydrogen-bond geometry (Å, º)

**Table:**

*D*—H···*A*	*D*—H	H···*A*	*D*···*A*	*D*—H···*A*
N1—H1*A*···O1^i^	0.888 (18)	2.021 (19)	2.8933 (15)	167.2 (15)
N1—H1*S*···O1^ii^	0.907 (18)	1.997 (19)	2.9024 (14)	176.0 (16)
N3—H3*S*···N2^iii^	0.915 (18)	2.125 (19)	3.0322 (15)	170.9 (15)
N3—H3*A*···N3^iv^	0.875 (17)	2.363 (17)	3.2083 (16)	162.7 (15)

Symmetry codes: (i) *x*, −*y*+1/2, *z*+1/2; (ii) −*x*, −*y*, −*z*; (iii) −*x*+1, −*y*+1, −*z*; (iv) −*x*+1, *y*+1/2, −*z*+1/2.
